# A Review on Luminescent Benzoselenadiazole Derivatives: Comparison with Benzothiadiazoles, Properties, Applications, and Trends

**DOI:** 10.1002/asia.202500004

**Published:** 2025-06-19

**Authors:** Brenno A. D. Neto, John Spencer, Alexandre A. M. Lapis

**Affiliations:** ^1^ Laboratory of Medicinal and Technological Chemistry, Chemistry Institute (IQ‐UnB), Campus Universitário Darcy Ribeiro University of Brasília Brasília, Distrito Federal 70910‐900 Brazil; ^2^ Molecular Sciences Graduate Program Universidade Estadual de Goiás Anápolis GO 75132–400 Brazil; ^3^ Sussex Drug Discovery Centre, School of Life Sciences University of Sussex Falmer Brighton BN1 9QJ UK; ^4^ Universidade Federal da Fronteira Sul Chapecó SC 89815–899 Brazil

**Keywords:** Benzoselenadiazole, Benzothiadiazole, Benzoxadiazole, Fluorescence, Heavy Atom, Imaging, Luminescent, Review

## Abstract

This review comprehensively analyzes key parameters that govern the use of luminescent benzoselenadiazole (BSD) derivatives. We examine the main factors affecting their photophysical properties, including structural variations, heavy atom effects and environmental conditions, and discuss their potential applications in bioimaging technology. Whenever possible, the properties were compared to those of 2,1,3‐benzoxadiazole and 2,1,3‐benzothiadiazole analogues. This review also highlights emerging trends and future directions in the research and development of BSD compounds, underscoring their significance and potential impact in advancing phototechnology and related fields. We also aim to offer valuable insight into the versatile nature of BSD derivatives and their expanding role in scientific and technological innovations.

## Graphical abstract

This review explores the photophysical behavior and bioimaging applications of luminescent benzoselenadiazole (BSD) derivatives, in comparison with benzothiadiazole (BTD) analogues. Emerging trends highlight BSDs as promising tools in advanced phototechnology.

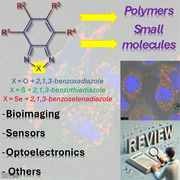

## 1 Introduction

**Figure 1:**
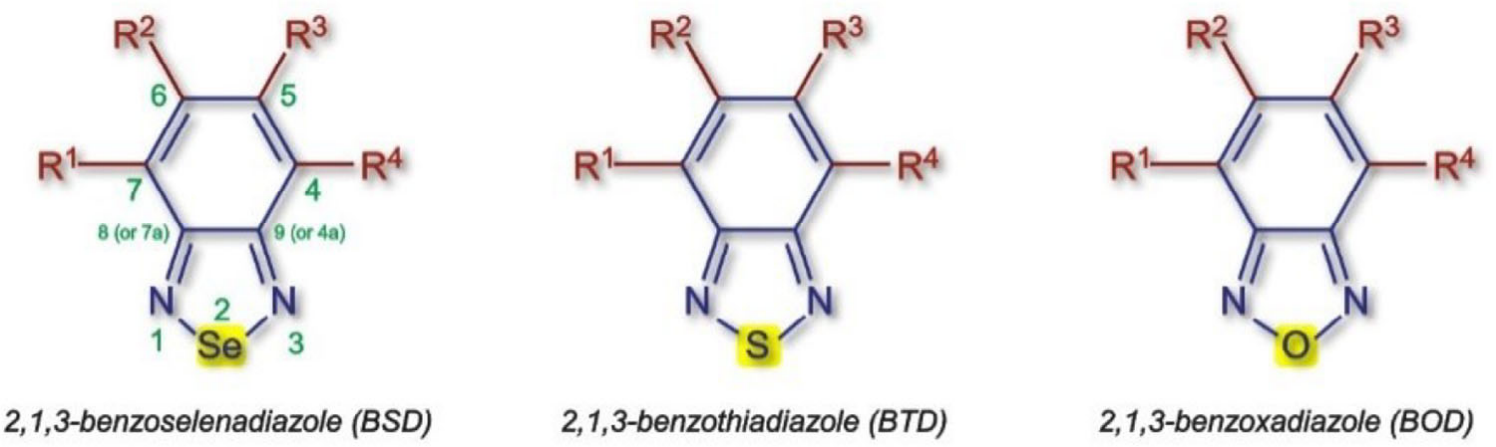
BSD, BTD, and BOD heterocycles.

2,1,3‐Benzoselenadiazole (BSD) is a Se‐containing heterocyclic compound with attractive luminescent properties (Figure 1) that is found preferentially in its 1,2‐quinoid form. Structurally, BSD derivatives incorporate a Se atom bound to the two nitrogen atoms in the five‐membered ring. Conversel, 2,1,3‐benzothiadiazole (BTD) and 2,1,3‐benzoxadiazole (BOD), respectively, incorporate a S and an O atom instead of Se (Figure 1).

The properties and applications of a widely used commercially available NBD (7‐nitro‐2,1,3‐benzoxadiazole), a BOD (Figure 1) derivative, have been reviewed elsewhere.^[^ ^1^ ^]^ NBD derivatives are also widely applied, especially in nucleophilic aromatic substitution reactions for the detection of reactive S moieties (e.g., H_2_S).^[^ ^2^, ^3^, ^4^, ^5^, ^6^ ^]^ Currently, fluorescent BOD derivatives are extensively used and may be applied, for example, as fluorescent live cell imaging probes,^[^ ^7^, ^8^, ^9^, ^10^ ^]^ among many other photo‐based applications.^[^ ^11^, ^12^, ^13^, ^14^, ^15^, ^16^, ^17^, ^18^, ^19^, ^20^, ^21^ ^]^

BTD heterocyclic derivatives (Figure 1) exhibit highly attractive physical and photophysical properties and are utilized in nearly all areas of luminescence.^[^ ^22^, ^23^, ^24^, ^25^ ^]^ Since 2010, BTDs have also been applied as a new class of bioimaging probes with great success, as highlighted elsewhere.^[^ ^26^ ^]^ Currently, more than fifty groups around the world have published work describing different bioimaging applications of this class of luminescent compounds, as recently reviewed elsewhere.^[^ ^27^ ^]^

**Scheme 1:**
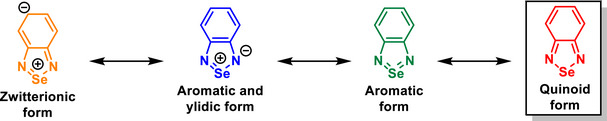
Possible BSD mesomeric forms. Note that all of these structures may contribute to the luminescent properties of BSD derivatives, although the 1,2‐quinoid form is likely the preferred and most abundant among these possibilities.

BSD derivatives (Figure 1), however, are far less explored than their chalcogen analogues in the field of luminescence. Their photophysical properties are complex to understand in fine detail, especially due to the significant contribution of the heavy atom (Se) effect. Despite this complexity, many of these observed properties are attractive for the application of such molecules in different areas of phototechnology. As we intend to explore in this review, fields such as OLEDs, solar cells, bioprobes, and others have benefited from the use of luminescent BSD derivatives. It has been known for many decades that BSDs can exist in four mesomeric forms (Scheme 1),^[^ ^28^ ^]^ each potentially contributing to the luminescent properties of these heterocyclics.

In this review, we analyze key parameters for the use of luminescent BSD derivatives, focusing on their unique properties and potential applications. We examine the main factors capable of influencing their photophysical properties, such as the heavy atom effect of Se, structural variations, and environmental conditions. Finally, we discuss emerging trends and future directions in the research and development of BSD‐based compounds, highlighting their significance and potential impact on advancing phototechnology and related areas. This comprehensive review aims to provide valuable insights into the versatile nature of BSD derivatives and their expanding role in scientific and technological advancements.

## 2 The Heavy Atom (Se) Effect

Heavier chalcogens, compared to their lighter counterparts, confer distinct physical properties due to fundamental differences such as atomic size, bond length, electronegativity, and polarizability.^[^ ^29^ ^]^ These variations significantly affect the morphology, crystallinity, and charge transport properties of the heavy atom‐containing derivatives.^[^ ^30^, ^31^ ^]^ Many heavier organochalcogens do not exhibit significant luminescence due to strong spin‐orbit coupling, which enhances intersystem crossing (ISC) to the triplet state, followed by non‐radiative relaxation.

**Scheme 2:**
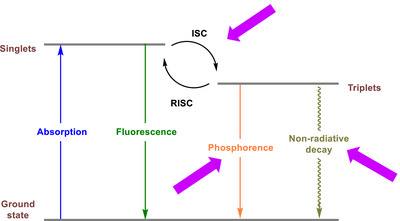
Representative photophysical pathways of heavy atom‐containing organic derivatives. Note that the presence of a heavy atom may also facilitate spin‐orbit coupling (SOC) and other non‐indicated non‐radiative decay processes. ISC = intersystem crossing and RISC = reverse intersystem crossing. The pink arrows indicate processes typically, but not always, favored by the presence of the heavy atom.

Replacing S with Se often induces significant changes in photophysical properties due to the heavy atom effect.^[^ ^32^ ^]^ Selenium's presence typically enhances spin‐orbit coupling (SOC), leading to a pronounced increase in the rate of ISC, which facilitates more efficient transitions between singlet and triplet states. This results in altered luminescence behavior, particularly by facilitating the occupation of triplet states and non‐radiative decay processes, thereby reducing light emission (Scheme 2). The presence of Se also accelerates internal conversion, further contributing to non‐radiative deactivation pathways.^[^ ^33^ ^]^ The delocalization effect induced by Se enhances luminescence intensity and stability due to its strong spin‐orbit coupling, which facilitates efficient ISC and enhances radiative decay pathways. The presence of Se extends the conjugation of the electronic system, lowering the energy gap between excited and ground states, while promoting a more stable excited‐state population. This delocalization reduces non‐radiative decay channels, improving both quantum yield and photostability, ultimately leading to more intense and prolonged luminescence.^[^ ^34^ ^]^ This effect has been clearly noted in stilbene derivatives applied in photoisomerization studies.^[^ ^33^ ^]^

Computational studies^[^ ^35^ ^]^ showed that Se's presence in some molecules facilitates charge migration and orbital angular momentum changes, particularly between the *p_z_* and *p_xy_* orbitals of Se, contributing to higher SOC matrix elements and improved phosphorescent emission compared to S‐containing analogs. This resulted in optimized photophysical properties, including elevated quantum yields and reduced non‐radiative decay​. In addition, due to its size and the presence of *d* orbitals, Se contributes to greater delocalization of electron density, which affects the energy gap between the HOMO and LUMO.^[^ ^34^ ^]^ This delocalization leads to favorable changes in electronic transition properties, enhancing both the intensity and stability of luminescent emissions. The synergistic interaction between selenium's *d* orbitals and the molecular framework underscores its role in optimizing luminescence.

The *d* orbitals of Se also play a key role in determining accurate geometries and enhancing dipole moments.^[^ ^36^ ^]^ These orbitals contribute to more precise molecular structure predictions by accounting for polarization effects, which are crucial for second‐row elements. While their energetic impact is relatively small, *d* orbitals significantly influence bond angles and lengths, thereby improving the accuracy of ab initio calculations.^[^ ^36^ ^]^ This effect is particularly evident in S‐ and Se‐containing molecules, where the inclusion of *d*‐functions in computational models yields better agreement with experimental data.

The introduction of Se also influences the structural rigidity of the molecule in its excited state, leading to faster triplet state depopulation and shorter triplet lifetimes compared to S‐containing analogs. As reviewed, similar effects are noted for Te‐containing compounds.^[^ ^37^ ^]^ These effects collectively reduce the overall luminescent efficiency of Se‐based derivatives, while enhancing non‐radiative processes, making the heavy atom effect a crucial factor in tuning the photophysical behavior of these compounds for specific applications.^[^ ^38^ ^]^ The altered luminescence behavior and faster non‐radiative decay processes can be advantageous or limiting depending on the application.^[^ ^39^ ^]^ Faster non‐radiative decay can be advantageous in various photochemical and biological applications, particularly in photodynamic therapy and structural biology. For instance, in this study of heavy‐atom‐substituted nucleobases,^[^ ^39^ ^]^ Se substitution in 6‐selenoguanine significantly increases the rate of triplet decay by 835‐fold compared to 6‐thioguanine. This rapid decay reduces the lifetime of the triplet state, thereby limiting the potential for harmful side reactions, such as unwanted DNA damage or long‐lived ROS (reactive oxygen species) generation. Additionally, in biological systems, fast triplet decay minimizes prolonged interactions with molecular oxygen, which can otherwise lead to cytotoxicity. In photodynamic therapy, shorter triplet‐state lifetimes can enhance selectivity by ensuring that the excited‐state energy is transferred primarily to targeted molecules rather than diffusing indiscriminately, thereby improving treatment precision and minimizing side effects.

In this context, for applications such as photodynamic therapy, the heavy atom effect is used to optimize triplet‐state formation, enabling more effective energy transfer to surrounding molecules, such as in the generation of singlet oxygen.^[^ ^40^ ^]^ This property is particularly valuable for targeting and damaging cancer cells.^[^ ^41^ ^]^ Replacing lighter atoms (like S) with heavier counterparts (like Se) often shifts the absorption spectrum into the visible region, allowing for deeper tissue penetration in medical treatments​.^[^ ^42^, ^43^, ^44^ ^]^

The enhancement of ISC between singlet and triplet states also plays a crucial role in processes such as thermally activated delayed fluorescence (TADF).^[^ ^45^ ^]^ The increased SOC in heavy‐atom‐containing compounds accelerates ISC, enabling more efficient triplet state population and facilitating transitions to the singlet state via reverse intersystem crossing (RISC). These properties are particularly useful in applications like organic light‐emitting diodes (OLEDs) and photodynamic therapy, where efficient triplet state utilization is essential. For example, some researchers have verified an approach for photoluminescence data analysis using Arrhenius and Marcus theories to explain the heavy‐atom effect on blue TADF materials. This approach provided experimental electronic data on spin‐flip transitions, with SOC constants being particularly useful for analyzing the TADF mechanism.^[^ ^46^ ^]^

Although these features are generally observed, a recent study revisited the heavy‐atom effect, specifically that of Se, in TADF.^[^ ^47^ ^]^ Traditionally, heavy atoms like Se are expected to enhance ISC and RISC rates (see Scheme 2) by increasing SOC. However, the study reveals that this enhancement is not universal and depends on molecular and orbital configurations. In the evaluated molecules, the delayed fluorescence lifetimes of Se‐containing compounds were longer than anticipated, highlighting the role of the spin‐vibronic coupling mechanism. This mechanism involves a proximate localized triplet state (^3^LE) facilitating RISC rather than direct SOC effects from heavy atoms like Se. These findings challenge the assumed universal role of heavy atoms in enhancing TADF properties and underscore the importance of considering the broader molecular context.

Aggregation‐induced emission (AIE) properties are directly influenced by the heavy atom effect, not only during the aggregation process but also in the photophysical properties of the aggregates. For example, the preparation of AIE functional single‐chain polymer nanoparticles (SCNPs) and their application in H_2_O_2_ detection through the intermolecular heavy‐atom effect has been described.^[^ ^48^ ^]^ In this case, Te‐containing amphiphilic polymers were introduced to exploit the heavy‐atom effect, acting as a switch for fluorescence. This allowed the detection of H_2_O_2_ by modulating fluorescence intensity. The interaction between the SCNPs and the heavy atoms, particularly Te, leads to changes in fluorescence due to the heavy‐atom effect, which promoted efficient SOC and facilitated ISC. The heavy‐atom effect played a crucial role in tuning the fluorescence properties, and its reversible behavior in response to oxidative stimuli is explored.

### 2.1 Effects of Se in Fluorescent Molecular Probes and Their Biological Properties

In this section, we selected a few examples of Se‐containing derivatives to illustrate the effects of incorporating this heavy atom and its biological impact. Although this section does not directly describe BSD applications, it is essential to highlight that many of these studies provided the foundation for designing and interpreting photophysical properties, biological responses, and ultimately guiding the application of BSD derivatives.

Given the unique properties of Se, its incorporation may significantly enhance the desired photophysical properties – such as fluorescence intensity, stability, and absorption efficiency – while also expanding the potential biological applications of these molecular probes. As expected, these enhancements are intimately associated with the intended application. It could lead therefore to more robust imaging capabilities and greater efficacy in biological contexts, such as targeted drug delivery or bio‐imaging.^[^ ^49^ ^]^ Consequently, the simultaneous improvements in both photophysical and biological characteristics increase the overall utility and scientific interest in using these Se‐based derivatives across a range of applications.^[^ ^50^ ^]^ In recent decades, the field of Se biology has emerged, highlighting selenium's role as a trace element in various biological systems. Since then, a range of organoselenium compounds has been developed, particularly to replicate redox transformations.^[^ ^51^, ^52^, ^53^ ^]^

In this context, the presence of Se (occasionally Te) atoms in molecular probes significantly enhances their performance in detecting biologically relevant analytes, primarily due to their unique redox properties and high reactivity.^[^ ^54^ ^]^ Se, in particular, plays a critical role in the development of reversible and selective probes. Its ability to undergo redox transformations, switching valence states between Se^2+^ and Se^4+^, enables selective detection of ROS and thiols through fluorescence “turn‐on” responses.^[^ ^55^, ^56^, ^57^ ^]^ This oxidation‐reduction capability mirrors biological processes, such as those in glutathione peroxidase, allowing the probes to emulate enzymatic functions that counteract oxidative stress. Additionally, selenium's electron‐donating properties help modulate the probe's electronic environment, facilitating targeted analyte recognition and enhancing photostability.^[^ ^58^ ^]^

**Scheme 3:**
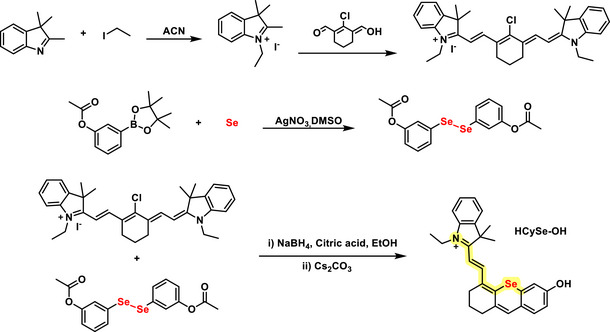
Synthetic route to **HCySe‐OH** (hemicyanine type) fluorescent dye. The overall yield was approximately 9%.

The substitution of O with Se (Scheme 3) in the hemicyanine dye scaffold (**HCySe‐OH** dye) significantly enhanced the photophysical properties and bioimaging capabilities. The introduction of Se extended both the absorption and emission wavelengths into the near‐infrared region, improving tissue penetration and signal‐to‐noise ratio, essential for in vivo applications.^[^ ^59^ ^]^ Additionally, Se increased the dye's singlet oxygen generation efficiency, crucial for applications like photodynamic therapy. These enhancements also translated to superior performance in bioimaging, particularly in cancer cells, where the Se‐substituted dye demonstrated strong fluorescence signals and efficient ROS generation under specific laser irradiation (760 nm), facilitating precise imaging and therapeutic effects.

The antibacterial effect of the Se‐substituted **HCySe‐OH** was also evaluated against *Staphylococcus aureus* under conditions with and without light irradiation. In the absence of light, the dye showed no significant inhibitory effects on bacterial growth, indicating that it does not exhibit inherent toxicity in the dark. However, upon 760 nm laser irradiation, a substantial antibacterial effect was observed. The ROS generated under light exposure effectively inhibited bacterial proliferation, demonstrating the photodynamic antibacterial properties of **HCySe‐OH**.

**Scheme 4:**
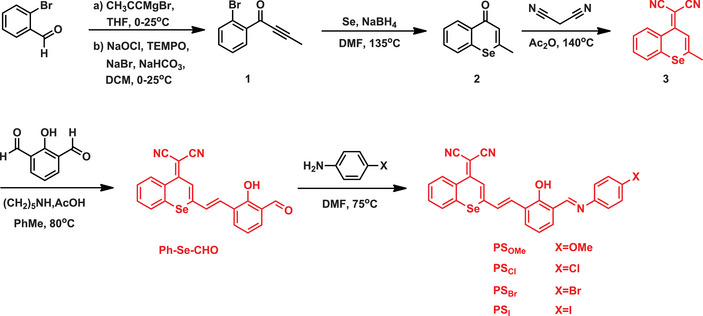
Synthesis of the Se‐containing **PSX** fluorophores.

Another study investigated a series of Se‐containing photosensitizers (named **PSX**) designed by substituting the O atom in benzopyran nitrile dyes with Se (Scheme 4).^[^ ^60^ ^]^ This modification leveraged the internal heavy‐atom effect, enhancing intersystem crossing efficiency and promoting ROS generation. The **PSX** derivatives demonstrated broad absorption (450 nm) and significant NIR fluorescence emission (650–800 nm), with excellent photostability under laser irradiation. In acidic, tumor‐like, hypoxic environments (pH 5.5–7.0), **PSX** dyes displayed remarkable stability. Cellular studies revealed efficient uptake in HeLa cells, resulting in NIR fluorescence imaging. Upon 532 nm laser irradiation, **PSX** fluorophores generated singlet oxygen and superoxide anions, triggering potent photodynamic effects with low dark toxicity. Phototoxicity tests confirmed over 90% cell death under light exposure, highlighting their potential for NIR‐guided imaging and photodynamic therapy applications of the Se‐containing derivatives.

**Scheme 5:**
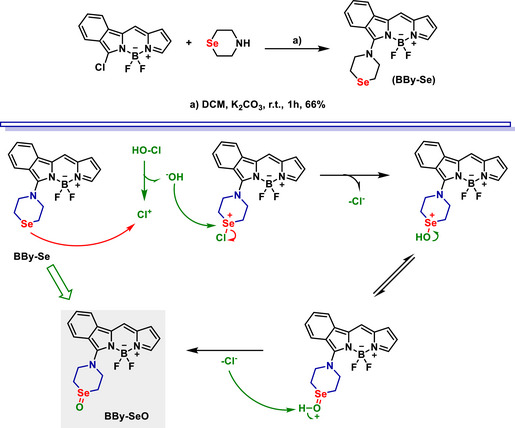
**BBy‐Se** synthesis (top) and sensing mechanism (bottom).

A Se‐based benzobodipy fluorescent probe, named **BBy‐Se**, was designed for the detection of hypochlorous acid (HOCl) in living cells and zebrafish (Scheme 5).^[^ ^61^ ^]^ The probe utilizes a PeT mechanism, where Se serves as the recognition site for HOCl. Upon oxidation of Se to selenoxide by HOCl, the PeT mechanism is inhibited, resulting in a strong fluorescence turn‐on response. **BBy‐Se** exhibits a Stokes shift of 82 nm, rapid response time (<10 s), and a low detection limit (≈ 11 nM), making it highly sensitive and selective for HOCl. The probe demonstrates excellent performance in bioimaging applications, showing bright fluorescence in HeLa cells and zebrafish. Additionally, its specificity was confirmed through negligible response to other reactive oxygen and nitrogen species, highlighting its practical utility for biological studies.

These aforementioned examples indicate the effects observed with the incorporation of Se in fluorescent derivatives and how the presence of this element influences the photophysical properties and biological responses of the tested dyes. In the next section, we will explore the use of BSD derivatives and the effects observed in the described structures.

### 2.2 BSD Derivatives in Their First Decade of Application

**Figure 2:**
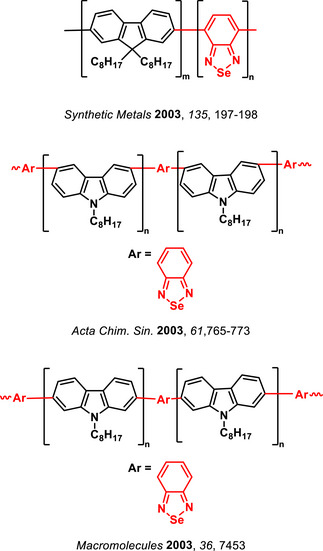
Polymeric structures incorporating the BSD heterocycle designed to tune the photophysical properties of polymers.

The use of BSD derivatives as fluorescent compounds dates back to the end of2003, when pioneering works were published incorporating the Se‐containing heterocycle into polymeric structures (Figure 2) to enhance their photophysical properties.^[^ ^62^, ^63^, ^64^ ^]^ In BSD‐containing units within carbazole‐based copolymers, the presence of this heavy atom enabled tuning of the band‐gap by leveraging the Se properties as energy traps and significantly enhancing energy transfer efficiency from wide band‐gap carbazole units. This mechanism allowed for efficient tuning of emission across the visible spectrum, showcasing the utility of Se in modifying the photophysical properties of the designed polymeric structures.

An effective intrachain energy transfer is observed when S (BTD‐containing) in similar heterocyclic compounds is replaced with Se (BSD‐containing) in carbazole‐containing copolymers (Figure 2), resulting in red‐shifted photoluminescence emissions due to selenium's larger atomic size and lower electronegativity.^[^ ^63^ ^]^ These properties enhanced intermolecular interactions, improving hole injection and light‐emission capabilities.

In fluorene‐based copolymers (Figure 2), the incorporation of BSD units narrowed the band gap, enabling red‐shifted photoluminescence, and enhancing electroluminescence efficiency. Selenium's larger size and unique electronic properties promote a substantial Stokes shift, rigidify the polymer chain, and enable efficient exciton confinement and emission within the Se‐containing units.^[^ ^64^ ^]^

The improved photophysical properties observed in these studies by incorporating the BSD core into polymeric structures served as the foundation for future polymeric developments. In a study published a few years later,^[^ ^65^ ^]^ a single polymer capable of emitting white light was developed by incorporating small amounts of orange‐light‐emitting BSD units into the backbone of a bipolar blue‐light‐emitting polyfluorene. Partial energy transfer from the blue‐fluorescent polyfluorene to the orange‐fluorescent component resulted in dual emissions from the copolymer, effectively combining the individual emissions of both emissive species. By adjusting the BSD content, white‐light emission was obtained. Tests in a light‐emitting device demonstrated that, due to the absence of phase separation in this covalently doped single‐polymer system, the color coordinates remained stable despite variations in the operating potential.

**Scheme 6:**
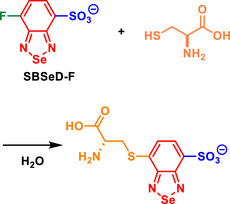
Cysteine reaction monitored via fluorescence using a BSD derivative. The reaction was performed in situ.

Also in 2003, the application of a small‐molecule BSD derivative to monitor its reaction with cysteine was reported (Scheme 6).^[^ ^66^ ^]^ The Se‐containing derivative exhibited unique photophysical behaviors compared to its S analog (BTD derivative). These derivatives were proposed as water‐soluble fluorescent reagents for thiols, particularly for derivatization in biochemical analyzes, emphasizing the reaction with this specific amino acid. Upon reacting, via an S_N_Ar with cysteine, BSD derivatives (named **SBSeD‐F**) displayed a maximum emission wavelength of 542 nm, which is red‐shifted compared to BTD derivatives (named **SBThD‐F**, 517 nm) and BOD derivatives (named **SBD‐F**, 514 nm). This shift highlighted the role of Se in extending the emission wavelength further into the visible region.

**Scheme 7:**
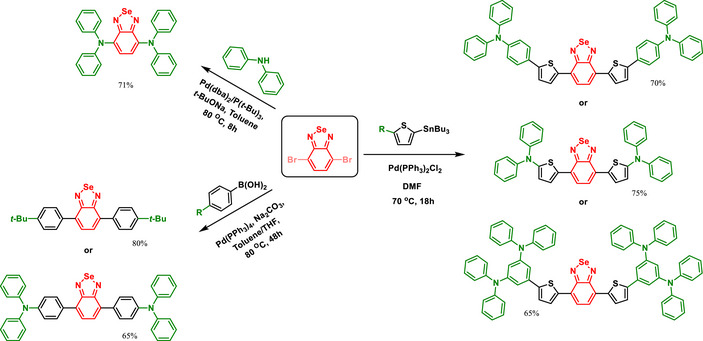
BSD‐containing diamine and tetramines derivatives.

A comprehensive study of BSD‐bridged amines focusing on their electrooptical properties revealed notable findings. A series of diamines and tetramines featuring the BSD heterocycle as a central bridge exhibited distinct absorption and emission characteristics influenced by the electron‐donating strength of the capping groups (Scheme 7).^[^ ^67^ ^]^ These compounds displayed a red‐shift in optical spectra compared to their thiadiazole analogs, attributed to the electron‐deficient nature of the BSD core. The authors proposed that the BSD core is more electron‐deficient than the BTD structure. Cyclic voltammetry revealed redox behaviors associated with the BSD core and the donor groups. The synthesized compounds exhibited lower oxidation potentials and more positive reduction potentials compared to analogous BTD derivatives, underscoring the impact of Se on electronic properties.

**Scheme 8:**
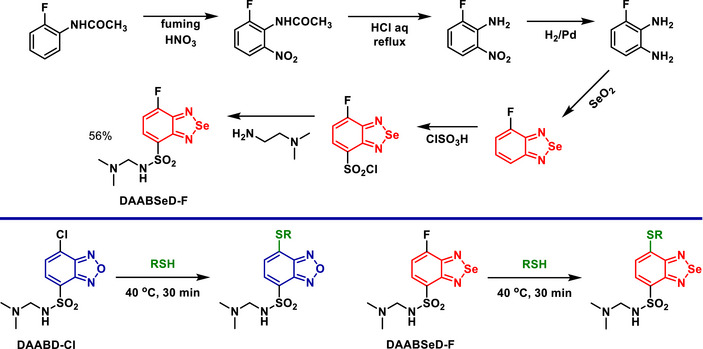
Synthesis of the BSD‐containing sensor (**DAABSeD‐F**) (top) for simultaneous detection of peptides or proteins (bottom) via a light‐up effect induced by C─S bond formation.

A new fluorogenic BSD derivative (named **DAABSeD‐F**) was developed for the simultaneous determination of peptides or proteins from different samples and was compared to a BOD derivative (Scheme 8).^[^ ^68^ ^]^ The BSD derivative exhibited distinct fluorescence properties, with excitation and emission wavelengths red‐shifted by approximately 30–40 nm compared to its BOD counterpart, named **DAABD‐Cl**. **DAABSeD‐F** demonstrated chemical selectivity, reacting exclusively with thiols, while non‐thiol compounds like alanine, serine, and tyrosine did not produce fluorescence upon reaction. The derivative was tested alongside **DAABD‐Cl** in proteomics workflows, enabling simultaneous labeling and detection of peptides or proteins under different conditions. This method facilitated quantitative comparisons and the identification of proteins from samples cultured under varying conditions, such as drug‐treated versus untreated cultures.

The successful application of **DAABSeD‐F** likely prompted the authors to synthesize new derivatives based on the molecular architecture of the BSD derivative. The new derivatives exhibited similar performance, leading to the development of a series of Se‐containing fluorogenic reagents.^[^ ^69^ ^]^

**Scheme 9:**
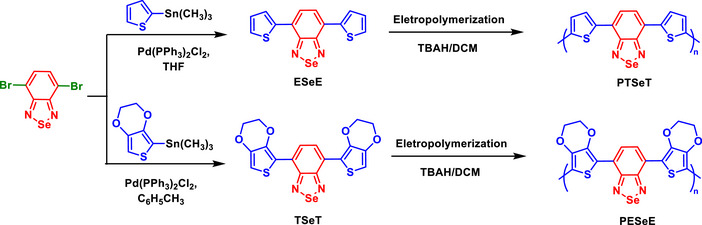
Synthesis of the monomers (**ESeE** and **TSeT**) and polymers (**PESeE** and **PTSeT**), which were subsequently compared with their BTD analogs.

A study explored the incorporation of Se into donor‐acceptor systems, specifically BSDs, in comparison to their S analogs, BTDs (Scheme 9).^[^ ^70^ ^]^ Se significantly influenced the properties of both the monomers and the resulting polymers, **PTSeT** and **PESeE**, by affecting their electronic, optical, and electrochemical characteristics. The Se atom in BSDs contributed to a lower electron affinity compared to BTDs, making the BSD core a less effective acceptor than the BTD. This difference was reflected in the redox behavior, as monomers containing Se exhibited higher cathodic reduction potentials, as shown by cyclic voltammetry data. These findings contrast with a previous suggestion that BSD might be more electron‐deficient than BTD.^[^ ^68^ ^]^ However, the study of **PTSeT** and **PESeE** marked a significant advancement in the development of neutral‐state green polymers, addressing a longstanding challenge in completing the RGB color spectrum for electrochromic applications.^[^ ^70^ ^]^ The polymer **PESeE**, featuring three distinct absorption bands, effectively absorbs in the red and blue regions of the spectrum, producing a vibrant green color in its neutral state.

The emissions resulting from the absorption in the blue and red bands are intrinsically linked to the electronic transitions in the polymeric electrochromic materials studied. **PTSeT**, which exhibits absorption bands at 350 nm (blue) and 600 nm (red), undergoes a transition that results in a deep‐red emission at 666 nm when dissolved in DMSO. This behavior suggests that the polymer efficiently absorbs high‐energy photons in the blue region, as well as lower‐energy photons in the red region, leading to radiative relaxation in the red spectral region. On the other hand, **PESeE**, which is the first reported neutral state green polymer bearing three well‐defined absorption bands at 34, 448, and 796 nm, exhibits a color transition from green to transparent sky blue upon oxidation. The red and blue absorption bands of **PESeE** effectively deplete simultaneously during oxidation, ensuring its electrochromic performance and potential application in optical displays. The presence of distinct absorption bands across the visible spectrum enabled fine‐tuning of the emitted color, highlighting the versatility of these polymers in electrochromic and emissive applications.

**Scheme 10:**
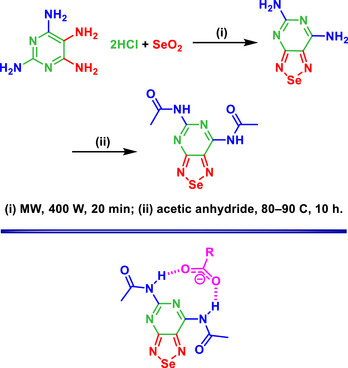
Synthesis of the fluorescent BSD small‐molecule (top) applied to aliphatic carboxylate detection (bottom).

A small molecule, based on a BSD‐fused pyrimidine receptor, was designed as a fluorescence sensor for the selective recognition of aliphatic monocarboxylates, such as acetate and pivalate (Scheme 10).^[^ ^71^ ^]^ The inclusion of Se in the heterocyclic core imparts unique photophysical properties to the compound, enhancing its performance as a selective and sensitive fluorescent probe. The receptor exhibits a bathochromic shift in its fluorescence emission upon interaction with monocarboxylates, attributed to strong hydrogen bonding facilitated by the Se atom. This shift is significant for detecting these specific anions and is evidenced by a measurable color change visible to the naked eye.

**Figure 3:**
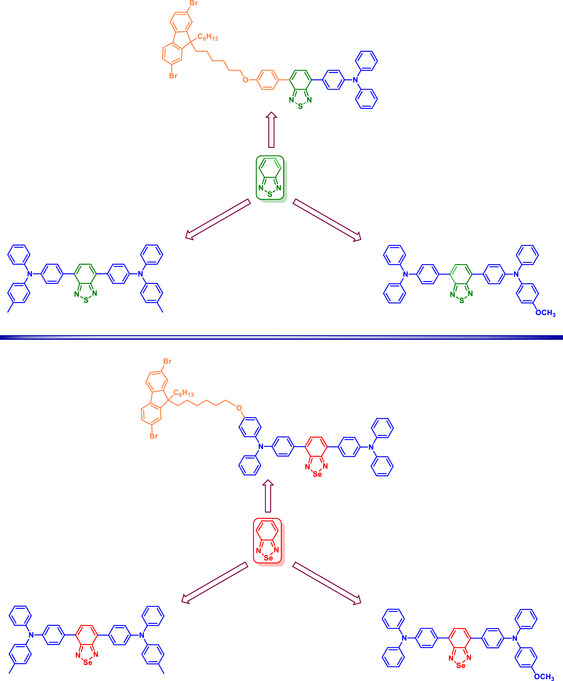
Examples of tested BTD and BSD derivatives with comparable photophysical properties.

Two studies showed that BSD derivatives exhibited distinct advantages over BTDs (naphthothiadiazoles in these studies)^[^ ^72^, ^73^ ^]^ due to their enhanced polarity, resulting in longer emission wavelengths. The Se atom's higher atomic number increased the SOC effect, influencing photophysical properties such as fluorescence quantum yield and emission wavelength. While BSD derivatives exhibited a smaller redshift compared to BTDs, they provided improved photostability and slightly higher efficiencies under specific conditions. In contrast, BTD derivatives benefited from extended conjugation through fused‐ring structures, which led to a more significant redshift in emission and higher quantum yields compared to traditional S compounds. For example, BTD‐based dopants displayed emission maxima up to 666 nm and quantum yields of approximately 0.35, whereas BSD derivatives reached a peak emission at 643 nm with quantum yields up to 0.51. These results suggested that BTD derivatives could achieve superior performance in applications requiring deeper red emissions, albeit with reduced quantum yields relative to BSD derivatives. Overall, BSD derivatives offered a balance between emission wavelength and efficiency, making them suitable for applications prioritizing energy transfer and stability, while BTDs excelled in applications requiring longer wavelengths and broader absorption profiles (Figure 3).

**Scheme 11:**
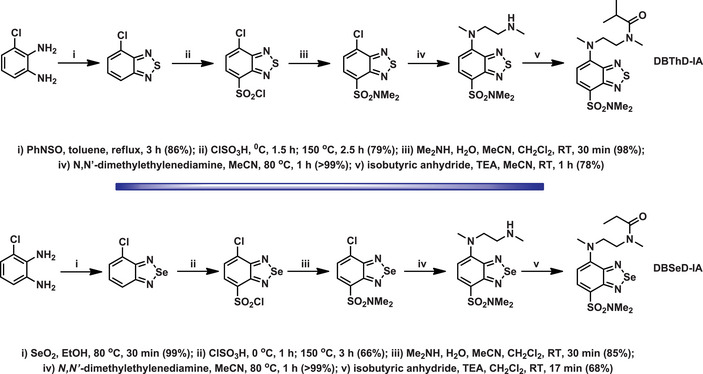
Synthesis of BSD and BTD derivatives as candidate components of a fluorescent polymeric thermometer.

An interesting study examined the properties of fluorophores named **DBThD‐IA** and **DBSeD‐IA**, comparing BSD‐ and BTD‐containing derivatives (Scheme 11).^[^ ^74^ ^]^ These fluorophores were environment‐sensitive, exhibiting fluorescence changes based on solvent polarity and hydrogen bonding. **DBSeD‐IA** displayed a significant bathochromic shift in both absorption and emission compared to the BTD‐based **DBThD‐IA** and O‐based (BOD‐containing) **DBD‐IA** derivatives. This shift was attributed to the Se atom's *d*‐orbital involvement, which extended conjugation and enabled lower energy transitions. **DBSeD‐IA** exhibited lower fluorescence quantum yields compared to **DBThD‐IA**, a result of the heavy atom effect of Se, primarily enhancing ISC and non‐radiative decay pathways. For instance, in water, **DBSeD‐IA** had a quantum yield of 0.0046, significantly lower than the 0.037 observed for **DBThD‐IA**. The emission wavelength of **DBSeD‐IA** in water was 672 nm, longer than the 616 nm of **DBThD‐IA**, highlighting its potential for near‐infrared applications. **DBThD‐IA** also demonstrated greater photostability compared to **DBSeD‐IA** and **DBD‐IA**, a critical attribute for applications such as intracellular temperature sensing, as shown with the **DBThD** nanogel.

**Scheme 12:**
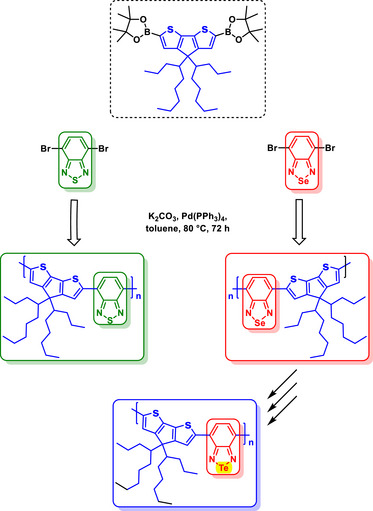
Synthesis of BSD, BTD, and Te‐containing polymers.

Polymeric BSD, BTD, and Te‐containing structures were systematically studied to assess the effects of substituting S, Se, and Te in donor‐acceptor (D–A) copolymers (Scheme 12).^[^ ^75^ ^]^ The substitution of heavier chalcogens (from S to Se to Te) resulted in a progressive red‐shift in the low‐energy absorption band, leading to narrower band gaps. The band gap values decreased from 1.59 eV (S) to 1.46 eV (Se) and further to 1.06 eV (Te). This trend was attributed to the lower ionization potential and increased bond lengths, which destabilized the LUMO and reduced the acceptor aromaticity, thereby affecting conjugation. The intensity of the low‐energy absorption band decreased as heavier atoms were incorporated, explained by the decreasing electronegativity of the chalcogen, which reduced the acceptor unit's ability to separate and stabilize charge. The high‐energy band remained relatively unaffected, as it was primarily centered on the donor unit. The Te‐containing polymers faced challenges in synthesis and exhibited lower photostability compared to their S and Se counterparts. In contrast, BSD‐based derivatives provided a balance between red‐shift and photostability, offering intermediate band gap narrowing and acceptable stability for practical applications. BTD‐containing polymers, however, displayed higher photostability and greater intensity in the low‐energy band, making them favorable for robust applications where stability was a key requirement.

**Scheme 13:**
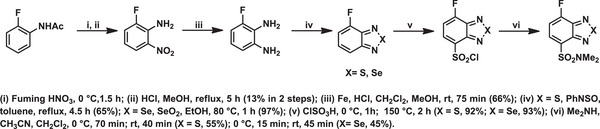
Synthesis of the environment‐sensitive BSD and BTD fluorophores via reactions with amines.

The development of fluorogenic reagents based on BTD and BSD derivatives for producing environment‐sensitive fluorophores via reactions with amines (Scheme 13) was disclosed.^[^ ^76^ ^]^ A comparative analysis of their properties highlighted both reagents (BTD and BSD precursors) were nonfluorescent but afforded fluorescent derivatives upon reacting with amines. The fluorescence proved to be environment‐sensitive, showing dependency on solvent polarity and hydrogen bonding. In nonpolar solvents (e.g., n‐hexane), fluorescence intensity is high, while it decreases in polar protic solvents such as water, indicating quenching effects. The quenching observed in the fluorescence of the BTD and BSD derivatives is attributed to the highly stable charge‐transfer state, which increased the probability of non‐radiative decay. The presence of an electron‐donating dimethylamino group and an electron‐withdrawing aromatic ring results in a strong ICT character in the excited singlet state. This leads to a decrease in the energy gap between the ground and excited states, thereby enhancing non‐radiative relaxation processes in accordance with the energy gap law.

The BSD dyes exhibit longer emission wavelengths and higher sensitivity to solvent polarity compared to the BTD analogue. A consistent bathochromic shift was observed for both dyes with increasing solvent polarity. For example, the emission wavelength of S‐containing dye shifted from 520 nm (n‐hexane) to 641 nm (water), while the Se‐containing molecule shifted from 573 nm (n‐hexane) to 688 nm (water). This behavior could be attributed to the ICT characteristics of their singlet excited states. As expected, the BTD‐based derivative showed approximately ten times higher photostability compared to related BSD fluorophores.

**Scheme 14:**
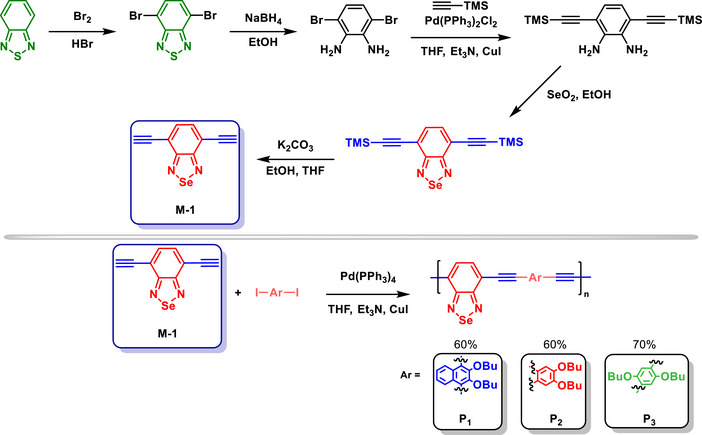
Synthesis of BSD‐containing polymers **P1**, **P2**, and **P3**.

The incorporation of BSD units significantly influenced the optical and electrochemical properties of studied D‐π‐A conjugated polymers (Scheme 14).^[^ ^77^ ^]^ Se, due to its larger atomic size and higher electron‐rich character compared to S, effectively lowered the band gap by reducing the LUMO energy levels. This modification resulted in red‐shifted absorption and emission properties, which enhanced the material's potential for applications in fluorescent devices. The BSD heterocycle facilitated the polymers' ICT, which optimized light absorption in the visible region and allowed for tunable electronic properties. Later, this group^[^ ^78^ ^]^ successfully applied these BSD‐based polymers as selective sensors for Ni(II) detection.

In a subsequent work of this group,^[^ ^79^ ^]^ similar polymers were also applied as sensors for Hg(II) ion detection. The larger atomic radius and lower electronegativity of Se compared to S provided distinct electronic properties, including bandgap reduction, which enhanced the optical and electronic features of the conjugated polymers. A bathochromic shift in both absorption and emission spectra, with the absorption peak extending to 477 nm and the emission to 576 nm, was observed in the BSD‐containing polymers. This redshift was attributed to the strong electron‐deficient nature of BSD, which effectively facilitated ICT processes between the heterocyclic structures and the fluorene donor

The soft nature of Se and its high affinity for soft acids like Hg(II) ions were critical for achieving the polymer's selectivity. The selective binding to Hg(II) b the BSD units disrupted the π‐conjugated system, resulting in fluorescence quenching and a pronounced “turn‐off” optical response. This interaction also contributed to the polymer's high sensitivity, with a detection limit of 1.9 × 10^−7^ mol L^−1^. The fluorescence quenching observed in the polymer sensor is primarily attributed to the intramolecular photoinduced charge transfer between the conjugated polymer backbone and the BSD‐Hg^2+^ complex, rather than solely due to a highly stable charge‐transfer state increasing the probability of non‐radiative decay. This quenching was characterized by a nonlinear Stern‐Volmer plot, indicating a mechanism beyond simple collisional deactivation. Instead, the strong interaction between the polymer's selenium‐containing BSD unit and Hg^2+^ resulted in a perturbation of the conjugated system, thereby altering the electronic structure and facilitating non‐radiative relaxation.

The negligible interference from other metal ions underscored the specificity of the Se coordination environment in the polymer. The study indicated that certain transition metal ions, including Pb^2+^, Co^2+^, Ni^2+^, Cd^2+^, Cu^2+^, Zn^2+^, Fe^3+^, and Ag^+^, exhibit some degree of fluorescence quenching. Among these, Ni^2+^, Co^2+^, and Ag^+^ showed slightly higher quenching effects compared to the others, but their influence remained significantly lower than that of Hg^2+^.

**Scheme 15:**
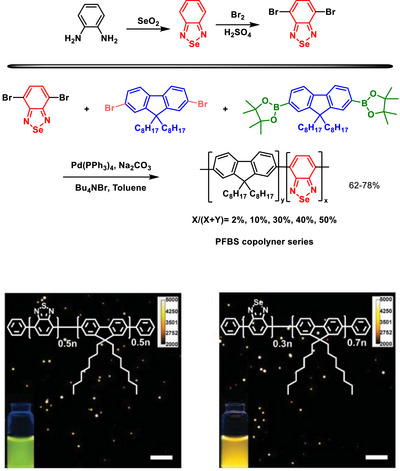
Synthesis of BSD‐containing **PFBS** copolymer series (top) and cell imaging application (bottom). Adapted with permission from Ref. [80]. Copyright 2013 Royal Society of Chemistry.

An interesting study explored the photophysical properties of BSD‐containing semiconducting polymer dots (Scheme 15).^[^ ^80^ ^]^ The BSD polymer, compared to its BTD analog, lowered the band gap, resulting in red‐shifted absorption and emission spectra. This change enhanced the brightness of the polymer dots, particularly at a 70:30 ratio of fluorene to BSD, which achieved an optimal quantum yield of 44%. Additionally, the incorporation of Se reduced self‐quenching effects through its electron‐rich nature, enabling higher fluorescence intensities even in condensed forms. The BSD‐based polymer dots exhibited superior photostability and brightness, maintaining performance over extended use. These properties made them highly effective for specific cellular labeling, with minimal nonspecific binding in biological imaging (Scheme 15).

**Scheme 16:**
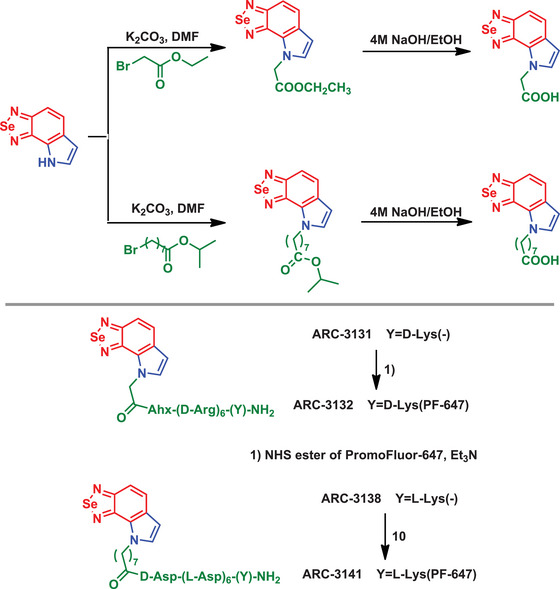
Synthesis of phosphorescent BSD‐containing peptides.

BSD‐containing protein kinase inhibitors were disclosed and evaluated for their ability to emit phosphorescence in the kinase‐bound state (Scheme 16).^[^ ^81^ ^]^ The presence of BSD in the molecules significantly influenced the photoluminescent properties of the probes, enabling long‐lifetime phosphorescence under specific conditions. This characteristic was predominantly observed when the probes formed complexes with protein kinases, highlighting the role of Se in stabilizing the triplet state of the luminophores. The BSD framework, compared to BTD and BOD derivatives, exhibited unique electron density and bonding attributes imparted by Se, which facilitated efficient FRET and enhanced signal amplification. The donor in the FRET mechanism is the BSD‐based phosphorescent luminophore, while the acceptor is the fluorescent dye PromoFluors‐647 (PF647). The BSD derivative acted as the energy donor due to its phosphorescence emission in the 500–700 nm region, whereas PF647 absorbs this energy and re‐emits it as amplified fluorescence, resulting in a significantly enhanced luminescence signal. The long‐lifetime emission signal, primarily on the microsecond scale, was strongly dependent on the interaction between the BSD core and the target protein. This interaction was essential for preventing quenching effects and achieving the desired luminescent output. In this study, BSD presence underscored the role of Se not only as a structural component but also as an active contributor to the functional optimization of the probes.

**Scheme 17:**
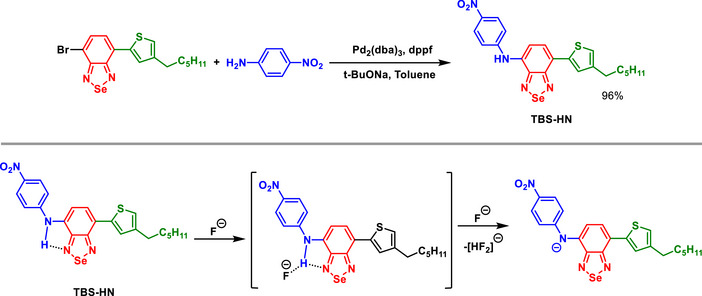
Synthesis of the selective BSD‐containing fluoride sensor and sensing mechanism.

The use of the BSD scaffold was pivotal in the development of the **TBS‐HN** sensor, which exhibited exceptional selectivity and sensitivity toward fluoride ions (Scheme 17).^[^ ^82^ ^]^ BSD significantly enhanced the sensor's photophysical properties, particularly by facilitating ICT and excited‐state intramolecular proton transfer (ESIPT) processes. These characteristics were attributed to heavy atom's electron‐withdrawing ability, which increased the acidity of the N─H proton and enabled precise deprotonation upon fluoride interaction. The resulting bathochromic shifts in the absorption spectrum and the unique ratiometric fluorescence signaling were directly linked to the BSD moiety. The distinct red‐to‐dark blue colorimetric change observable to the naked eye underscored the role of BSD in modulating the electronic transitions within the designed structure. Subsequent studies revisited the photophysical properties of this selective sensor using DFT calculations,^[^ ^83^ ^]^ concluding that the ESIPT process is unlikely to occur in such structures.

### 2.3 BSD Derivatives and New Developments

These aforementioned works demonstrated the versatility, utility, and tunable photophysical properties of BSD‐containing small molecules and polymeric fluorescent structures. They contributed to establishing and consolidating fluorescent BSDs as an important class of fluorophores with diverse applications and potential. In this section, we evaluate the progress observed following the publication of these previously cited articles. Many of these applications and advancements were possible because, after the initial decade, the behavior and properties of BSD‐based fluorophores were relatively well‐established, though there remains significant room for further development.

**Figure 4:**
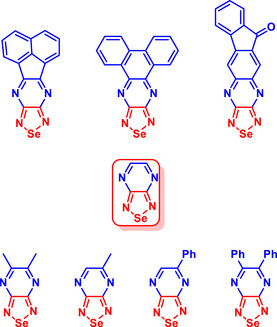
BSD‐pyrazine derivatives that afford persistent radical anions.

The incorporation of Se played a crucial role in enhancing the electron affinity of synthesized BSD‐pyrazine hybrid structures (Figure 4),^[^ ^84^ ^]^ as demonstrated by both experimental and theoretical data. The Se atom contributed to the stabilization of the persistent radical anions generated during electrochemical reduction. BSD presence facilitated the formation of thermodynamically stable radical species, which show promise for applications in magnetically active functional materials, as pointed in the work. Additionally, the influence of Se on molecular geometry and electronic distribution was pivotal to the observed stability and reactivity of these heterocycles, as confirmed through DFT calculations and EPR spectroscopy.

Following this work, a new study highlighted the comparative properties of benzochalcogenadiazole derivatives containing Se and Te,^[^ ^85^ ^]^ focusing on their redox behavior and structural characteristics. Both elements enhanced the electron‐accepting ability of the compounds, as demonstrated by cyclic voltammetry and supported by DFT calculations. Te, with its higher atomic number, exhibited a stronger electron‐accepting capacity compared to Se, contradicting trends in electronegativity and atomic electron affinity. The formation of radical anions was a key feature studied, with Se derivatives producing persistent radical anions stabilized by charge delocalization. In contrast, Te compounds exhibited greater instability under electrochemical conditions, leading to the formation of novel anionic complexes with coordinated Te–Te bonds. These complexes were structurally characterized, and the Te–Te interactions were analyzed through QTAIM and DFT calculations, revealing partially covalent bonding. While the study did not describe photophysical properties, such as absorption or emission, it highlighted the distinct influences of Se and Te on electronic structure and stability. Te's heavier atomic number also contributed to significant spin‐orbit coupling effects, evidenced by *g*‐factor shifts in EPR spectroscopy, further differentiating it from Se. These findings underscored the complementary roles of Se and Te in tuning the electronic and structural properties of benzochalcogenadiazole derivatives.

**Scheme 18:**
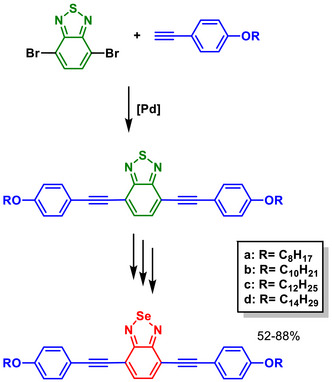
Synthesis of BSD‐containing liquid crystals.

The incorporation of BSD significantly influenced the photophysical properties of the synthesized liquid crystal derivatives (Scheme 18).^[^ ^86^ ^]^ It induced a bathochromic shift in both absorption and emission wavelengths compared to their BTD‐containing counterparts, with emission maxima reaching the red region. The presence of Se enhanced ICT, leading to larger Stokes shifts and solvatochromic behavior. Additionally, BSD affected the fluorescence quantum yields, which, although slightly reduced due to the heavy atom effect, remained moderate and suitable for applications in light‐emitting devices.

**Scheme 19:**
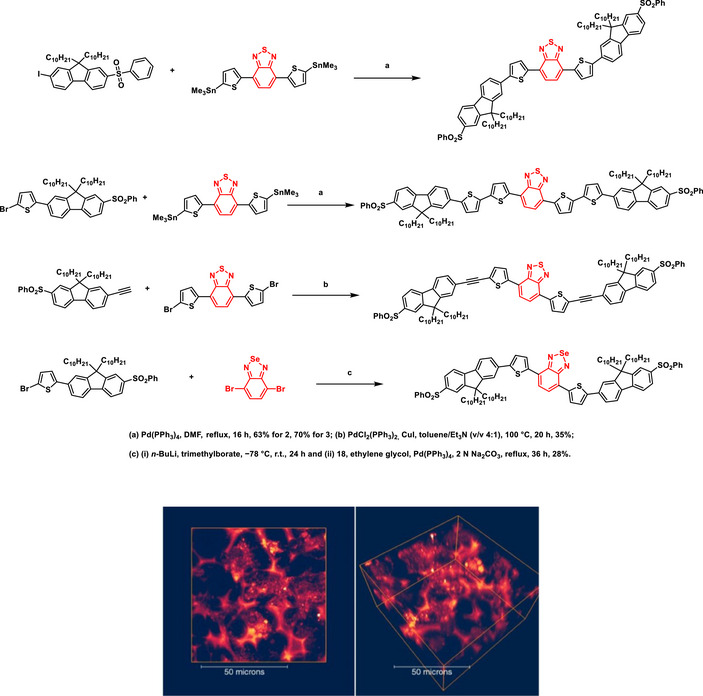
Synthesis of near‐infrared fluorescent two‐photon‐absorbing BSDs (top) and bioimaging application (bottom). Two‐photon fluorescence microscopy (2PFM) images of 3D‐cultured 3T3 cells stained with dye number 4 (10 µM), presented in the X−Y top‐down view (left) and the 3D side view (right). Adapted with permission from Ref. [87]. Copyright 2020 American Chemical Society.

One area of great interest that was poorly explored in the use of fluorescent BSD derivatives is bioimaging. Following the pioneering studies, a few additional examples were published, opening new possibilities in the imaging field using BSDs. One such work disclosed the use of π‐conjugated BSD derivatives as two‐photon probes for imaging 3T3 cells (Scheme 19).^[^ ^87^ ^]^ The incorporation of BSD enhanced fluorescence emission toward the near‐infrared region, with a notable bathochromic shift compared to their BTD analogs. This shift enabled deeper tissue imaging capabilities, essential for biological applications. Additionally, the use of BSDs improved fluorescence quantum yields and maintained high two‐photon absorption cross‐sections, optimizing the figure of merit for two‐photon fluorescence imaging.

**Scheme 20:**
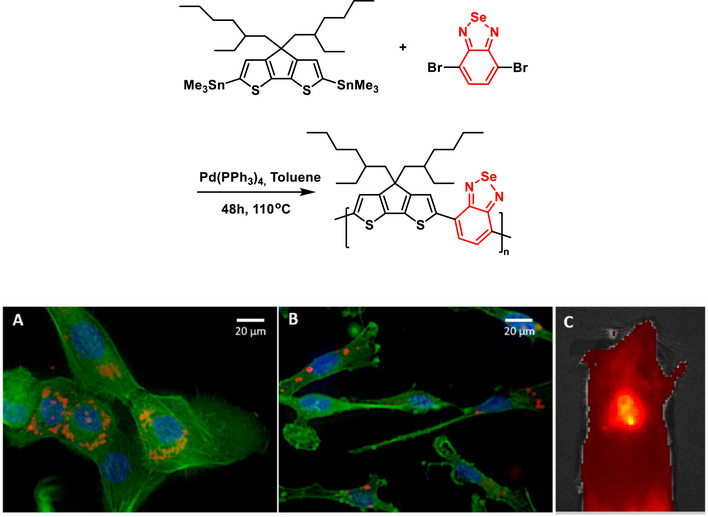
BSD‐containing polymeric nanoparticles of variable molecular weight synthesis (Top) named **PCPDTBSe** and imaging application (bottom). (A) MCF10A and (B) MDA‐MB‐231 cells were stained with Alexa Fluor 488‐phalloidin to visualize the cytoskeleton (green) and DAPI to label the nuclei (blue), showing the internalization of VMWNPs (red) near the perinuclear region in both cell lines. (C) VMWNPs with a 20:1 oligomer MW to high‐MW **PCPDTBSe** ratio were injected subcutaneously into a mouse and detected using IVIS imaging. Adapted with permission from Ref. [88]. Copyright 2020 American Chemical Society.

The inclusion of BSD in the polymeric backbone of the so‐called **PCPDTBSe** significantly influenced the photophysical properties and biological responses of the resulting nanoparticles (Scheme 20).^[^ ^88^ ^]^ The presence of BSD enhanced the NIR fluorescence emission of the low‐molecular‐weight oligomer fractions, which exhibited aggregation‐induced red‐shifted fluorescence upon nanoparticle formation. This effect resulted in a large Stokes shift of 275 nm, improving the signal‐to‐background ratio for imaging applications and minimizing tissue autofluorescence interference. In terms of biological response, BSD‐containing nanoparticles with variable molecular weights (VMWNPs) demonstrated excellent biocompatibility and stability in physiological environments. They were non‐cytotoxic to both normal (MCF10A) and cancerous (MDA‐MB‐231) cells in the absence of laser stimulation. Upon near‐infrared (800 nm) laser irradiation, the BSD‐based nanoparticles efficiently converted light into heat, leading to the photothermal ablation of triple‐negative breast cancer cells. Thus, these structures exhibited dual functionality, combining fluorescence imaging and photothermal therapy.

**Scheme 21:**
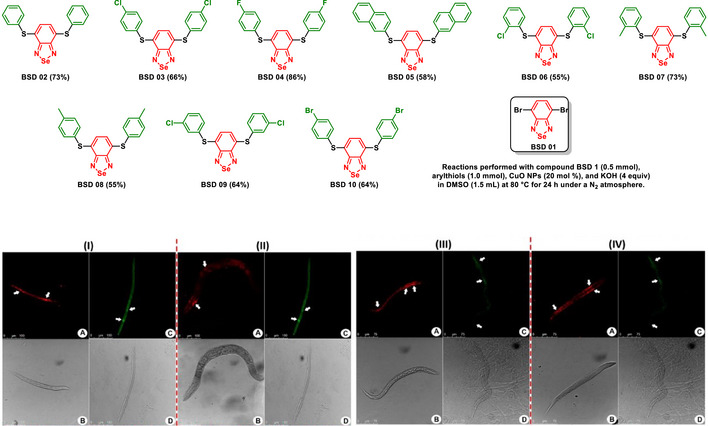
Small‐molecule BSD derivatives applied to stain lipid droplets selectively. Tissue staining in *C. elegans* using BSD dyes (1.0 µM) and BODIPY (12 µM) was conducted to compare BODIPY with (I) **BSD 02**, (II) **BSD 03**, (III) **BSD 07**, and (IV) **BSD 08**. (A) Displays the fluorescence emission of BSD dyes in the red channel, while (B) shows the fluorescence emission of BODIPY in the green channel. (C) and (D) also illustrate the normal morphological features of the samples captured through phase‐contrast microscopy. Reference scale bars are 75 and 100 µm. Adapted with permission from Ref. [89]. Copyright 2020 American Chemical Society.

Some of us disclosed the synthesis of a series of fluorescent BSDs applied as selective cell‐imaging probes (Scheme 21).^[^ ^89^ ^]^ BSD, as the basic framework, was responsible for the observed enhancement of ICT processes within the molecules, as evidenced by significant red‐shifts in their emission spectra, particularly in lipophilic environments. This improvement in photophysical behavior was attributed to the unique electronic characteristics of the BSD heterocycle, which contributed to greater stability under light irradiation and larger Stokes shifts, facilitating efficient imaging applications. Biologically, the synthesized BSD derivatives demonstrated remarkable selectivity and fluorescence intensity when staining lipid droplets in live cells and complex multicellular models like *Caenorhabditis elegans*. The developed BSD probes were effective at nanomolar concentrations, surpassing commercially available dyes like BODIPY in sensitivity and specificity. Notably, substituting S with Se enhanced lipid‐specific staining capabilities, highlighting the BSD influence in generating biologically relevant and photostable imaging probes.

A breakthrough contribution was disclosed by Alves and co‐workers.^[^ ^90^ ^]^ In this work, structures similar to those shown in Scheme 21 were synthesized, but the BTD core was replaced with the BSD heterocycle. The photophysical properties generally exhibited a slight redshift and larger Stokes shifts while maintaining similar molar extinction coefficients. This suggests that the arylselanyl substituents did not play a significant role compared to the acceptor moiety of the structures, that is, the BTD and BSD heterocycles.

**Scheme 22:**
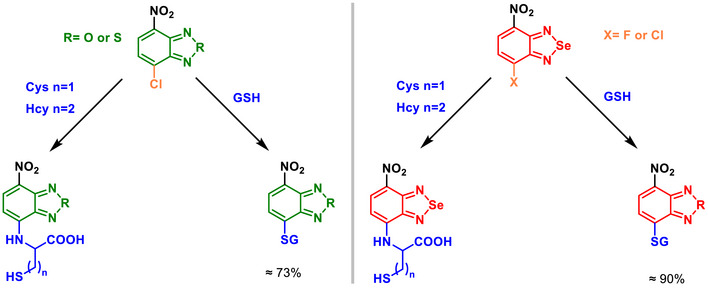
BOD, BTD and BSD derivatives applied to selectively detect distinct thiols (Cys/Hcy at 600 nm and GSH at 536 nm).

An article demonstrated the effects of BSD substitution for BTD or BOD on the photophysical properties and biological responses of fluorescent probes designed for the detection of biothiols (Scheme 22).^[^ ^91^ ^]^ BSD substitution in the probe significantly enhanced fluorescence properties by enabling dual‐channel emission to detect distinct thiols (Cys/Hcy at 600 nm and GSH at 536 nm). Compared to its BOD and BTD analogs, the BSD‐containing fluorophore reduced the HOMO‐LUMO gap, resulting in a bathochromic shift and an extended emission range, making it suitable for advanced imaging applications. Biologically, as indicated previously, Se‐containing probes exhibited high selectivity and rapid response times in detecting endogenous and exogenous thiols in HepG2 cells, with distinct fluorescence channels for Cys/Hcy and GSH. The probes also exhibited low cytotoxicity and excellent stability within physiological pH ranges, ensuring their potential applicability in studying thiol‐related metabolic pathways and diseases.

**Scheme 23:**
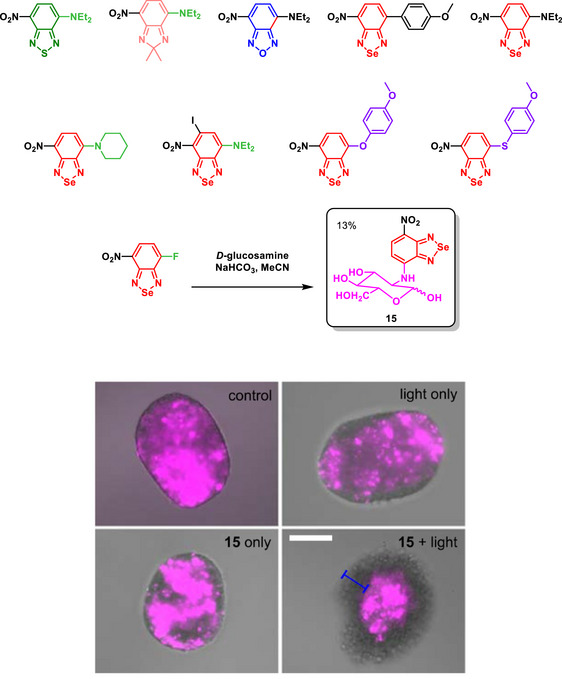
Amino‐substituted BSDs (and analogues) photoactivatable probes (top) for photodynamic activities in vitro and in vivo (bottom). Representative merged brightfield and fluorescence microscopy images (from three independent experiments) of U87‐nlsCrimson spheroids, with live cells expressing E2Crimson fluorescent protein (λ_em_ 645 nm, magenta). Spheroids treated with compound **15** combined with light exposure exhibited a ∼50 µm diameter circumference of dead cells (indicated by the blue bar). Scale bar: 100 µm. Adapted with permission from Ref. [92]. Copyright 2021 Springer‐Nature.

A breakthrough work investigated the photophysical properties and biological responses of amino‐substituted BSDs, emphasizing the role of Se (Scheme 23).^[^ ^92^ ^]^ Se in the BSD framework enhanced singlet oxygen generation due to its important spin‐orbit coupling capabilities, which were five times greater than those of other heteroatoms, facilitating efficient intersystem crossing. This feature enabled the production of cytotoxic ROS upon light irradiation, a key factor in photodynamic therapy. BSDs exhibited extended absorption wavelengths (480–565 nm) suitable for visible light activation, with negligible dark toxicity and near‐complete cell ablation post‐irradiation. The BSDs displayed high specificity and efficacy in targeting metabolically active cells. In bacterial applications, *D*‐amino acid‐conjugated BSDs selectively incorporated into bacterial peptidoglycan and triggered singlet oxygen production under light, achieving precise antimicrobial effects without damaging surrounding healthy cells. In cancer models, glucose‐derivatized BSDs targeted glioblastoma cells via GLUT transporters, resulting in selective cell death upon illumination. In vivo, BSDs eliminated glioblastoma microtumors in zebrafish models with minimal side effects, demonstrating both efficacy and safety.

**Figure 5:**
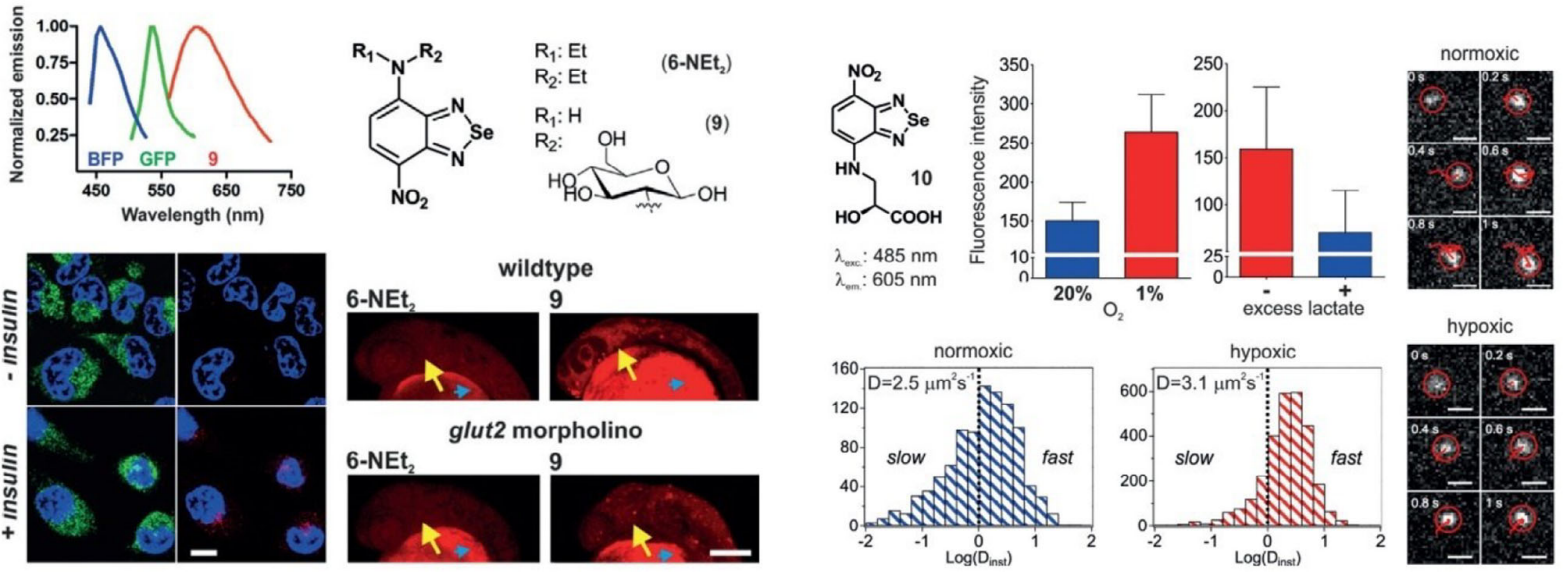
(Left) Fluorescence emission spectra of BFP (blue), GFP (green), and compound **9** (red). Uptake of compound **9** in GLUT4‐EGFP HeLa cells. Chemical structures of compounds **6‐Net_2_** and **9**. In vivo imaging of zebrafish embryo heads (28 h post‐fertilization, hpf) following the injection of **6‐Net_2_** or **9** into the yolk sac (indicated by blue arrowheads). Fluorescence images were captured from wild‐type zebrafish and zebrafish injected at the one‐cell stage with 4.2 ng of anti‐sense glut2 morpholino. Yellow arrows indicate the midbrain and hindbrain regions within the zebrafish embryo heads. Scale bar = 100 µm. (Right) Normoxia = blue and hypoxia = red condition. Tracking of fluorescent particles in untreated and DMOG‐treated HeLa cells after incubation with **10**. Scale bars = 1 µm. Adapted from Ref. [93]. Figure of public domain under the terms of the Creative Commons CC BY license.

Vendrell and co‐workers had also investigated the photophysical and biological properties of BSD derivatives, specifically compounds **6** and **10** (Figure 5), as part of their development of the so‐called SCOTfluors‐small, conjugatable, orthogonal, and tunable fluorophores for in vivo imaging.^[^ ^93^ ^]^ Compound **6**, a nitro‐BSD derivative, exhibited red fluorescence with an emission peak around 605 nm and a significant Stokes shift, enabling dual imaging applications. When conjugated to 2‐deoxyglucosamine (compound **9**), it effectively tracked glucose uptake in HeLa cells and zebrafish embryos, demonstrating selective transport via GLUT4 and GLUT2 transporters. Meanwhile, compound **10**, derived from nitro‐BSD and *L*‐isoserine, functioned as a red‐emitting fluorescent probe to study lactic acid metabolism. It showed increased uptake in hypoxic cancer cells compared to normoxic ones, correlating with lactic acid accumulation under low oxygen conditions. Flow cytometry and competition assays confirmed its transport through a common lactic acid pathway.

**Figure 6:**
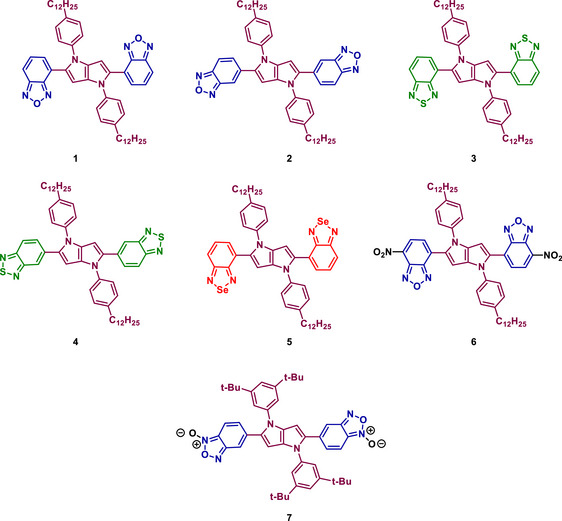
Structures of BOD‐, BTD‐, and BSD‐containing quadrupolar tetraarylpyrrolo[3,2‐*b*]pyrroles. Adapted from Ref. [94]. Figure of public domain under the terms of the Creative Commons CC BY license.

A recent work examined the impact of electron‐deficient heterocycles (BOD, BTD and BSD derivatives) on the photophysical properties of quadrupolar 1,4‐dihydropyrrolo[3,2‐*b*]pyrroles (DHPPs), a class of centrosymmetric chromophores with tunable optical responses (Figure 6).^[^ ^94^ ^]^ The findings revealed that incorporating these strongly electron‐withdrawing heterocycles at positions 2 and 5 of the DHPP core induces a substantial red‐shift in both absorption (470–620 nm) and emission (500–720 nm). Computational studies further elucidated the role of charge transfer processes in determining the fluorescence properties of these DHPP derivatives. While internal conversion was identified as the dominant non‐radiative deactivation pathway, ISC also contributed to fluorescence quenching in certain cases. The incorporation of BSD in the DHPP core, specifically in compound **5**, had a pronounced impact on its photophysical properties. Compared to its BOD and BTD‐containing analogs, the BSD‐substituted derivative exhibited a significant bathochromic shift in both absorption and emission spectra, with an emission maximum extending into the near‐infrared region. However, this red‐shifted emission was accompanied by a substantial decrease in fluorescence quantum yield, indicating that Se introduction enhances charge transfer characteristics but also increases non‐radiative decay pathways.

A few other studies had, in general, similar observations and applications as those already discussed in this work, but they have certainly contributed to extend the application of BSD fluorescent derivatives, as summarized in Table 1.

**Table 1:** Fluorescent BSD‐derivatives and their applications.

BSD Structure Type	Comment	Ref.
Highly conjugated π‐extended small molecule	The unorthodox chalcogen bonding in BSD‐based fluorophores enabled precise and selective molecular recognition, enhancing the detection sensitivity of trimethyl arsine vapor.	^[^ ^95^ ^]^
Conjugated π‐extended small molecule	BSD fluorophores exhibited reversible visible‐to‐NIR spectroscopic switching, mediated by electrochemical and solvent (solvatochromic) effects, enabling their application in electrochromic and optoelectronic devices.	^[^ ^96^ ^]^
Conjugated π‐extended small molecule	A BSD‐based compound exhibited unique *J*‐to‐*H* aggregation switching and was applicable in generating white light emission through co‐assembly with 1‐pyrenemethanol, showing potential for advanced optical materials.	^[^ ^97^ ^]^
Conjugated π‐extended small molecule	Symmetrical and unsymmetrical BSD red emitters exhibited significant biomolecular interactions, particularly with DNA and human serum albumin, suggesting potential applications in bioimaging and optical materials.	^[^ ^98^ ^]^
Polymeric BSD derivative and nanoparticulate system with PEG	Photothermal BSD‐containing polymer nanoparticles successfully interfaced with bacteria associated with kidney stones and, when stimulated with NIR light, generated localized heat capable of significantly reducing bacterial colonies, presenting a potential strategy for treating stone‐associated infections and minimizing risks like urosepsis.	^[^ ^99^ ^]^
Conjugated π‐extended small molecule applied in supramolecular polymerization	Small‐molecule fluorescent BTD and BSD derivatives were utilized in a supramolecular polymerization strategy with pillar[5]arene to transform dye aggregation from parallel to head‐to‐tail alignment, effectively suppressing aggregation‐caused quenching and enhancing solid‐state emission.	^[^ ^100^ ^]^
Conjugated homopolymers	Thieno[3,2‐b]thiophene‐based homopolymers were obtained using electron acceptor moieties such as BSD, thienopyrrolodione, and isoindigo, demonstrating tunable optoelectronic properties with applications in electrochromic devices and organic electronics.	^[^ ^101^ ^]^
Conjugated π‐extended small molecule	D–A–D type phenyl‐capped benzazoles with varied central heteroatoms (N, O, S, Se) exhibited tunable photophysical and electrochemical properties, including high quantum yields and emission color variation. Se had a pronounced effect on the band gap and emission properties.	^[^ ^102^ ^]^
Conjugated π‐extended small molecule	An efficient synthetic method for arylation at the C4/C7 positions was developed. The presence of Se in π‐extended BSDs enhanced charge‐transfer characteristics and red‐shifted photophysical properties compared to BTDs.	^[^ ^103^ ^]^
Conjugated π‐extended small molecule	The 4‐amino‐BSD moiety played a critical role in enhancing the regioselectivity of palladium‐catalyzed arylation of unactivated C(sp^3^)‐H bonds. It functioned as part of a bidentate directing group, enabling efficient C‐H activation and functionalization under mild reaction conditions.	^[^ ^104^ ^]^
Conjugated π‐extended small molecule	The BSD unit, compared to its BTD analog, contributed to increased molecular polarization and stronger intramolecular charge transfer, resulting in lower band gaps and red‐shifted absorption and emission.	^[^ ^105^ ^]^
Conjugated polymers	The CP backbone, primarily composed of phenylene units with a smaller proportion of BSD, allowed the CPs to emit distinct fluorescence colors depending on their state (solution or solid). This variation arose from differences in intermolecular electron transfer within the CP backbones.	^[^ ^106^ ^]^
Conjugated polymers	The electron‐withdrawing ability of the water‐soluble BSD‐containing conjugated polymers increased their sensitivity and selectivity in fluorescent sensors for Fe^3+^ and Hg^2+^ ions. This property enabled unique "turn‐on" and "turn‐off" fluorescence mechanisms.	^[^ ^107^ ^]^
Conjugated polymers coated with hyaluronic acid	BSD was incorporated into the polymer backbone of photothermal nanoparticles coated with hyaluronic acid, improving their NIR absorption and photothermal conversion efficiency compared to the BTD analogue. This enabled targeted photothermal ablation of colorectal cancer cells in 3D tumor organoids.	^[^ ^108^ ^]^
Conjugated polymers	The BSD exhibited enhanced electron‐acceptor strength compared to BTD derivatives, resulting in a pronounced ICT effect, a lower HOMO‐LUMO gap, and significant solvatochromic behavior. Additionally, Se in the side substituents enabled dimerization via S/Se···N interactions.	^[^ ^109^ ^]^
Conjugated π‐extended small molecule	TD‐DFT was applied to investigate ISC in BSD‐based D–A–D molecules. The Se heavy atom effect significantly enhanced SOC, facilitating ISC and increasing triplet yields. The findings highlighted the ability of TD‐DFT to predict ISC trends effectively, providing insights into fluorescence and triplet quantum yields.	^[^ ^110^ ^]^

## 3 Summary and Outlook

Especially over the past two decades, BSD derivatives have emerged as a versatile class of fluorophores with unique photophysical properties driven by the heavy atom effect of Se. This review has highlighted the structural and electronic contributions of BSD to fluorescence‐based applications, including bioimaging, photodynamic therapy, and optoelectronics. Key advancements include the development of BSD‐based polymeric materials with enhanced energy transfer efficiency, selective probes for biologically relevant analytes, and fluorogenic sensors with remarkable sensitivity and specificity. When possible, a straight comparison with BTD analogues was also discussed.

Despite these significant achievements, challenges remain in optimizing BSD derivatives for broader applications. The heavy atom effect, while advantageous for certain uses, limits fluorescence quantum yields and introduces complexities in photophysical behavior. Future research should focus on mitigating these limitations through molecular architecture strategies that balance radiative and non‐radiative decay processes.

Emerging trends, such as two‐photon fluorescence imaging and aggregation‐induced emission, highlight the potential of BSD derivatives in next‐generation phototechnology. The incorporation of Se into D–A systems continues to expand the tunability of electronic and optical properties, offering opportunities for innovation in energy conversion devices, environmental sensing, and theranostics.

As the reader has likely noted, although BSD‐containing small‐molecule synthesis and applications have increased in the last decade, the use of this important heterocycle is predominantly in polymer technology. Therefore, a vast avenue of opportunities remains to be explored in the design, synthesis, and light‐emitting applications of small‐molecule BSD compounds, particularly in bioimaging, where only a few studies have been reported.

In conclusion, BSD derivatives stand at the frontier of fluorophore design, with untapped potential to advance both fundamental research and practical applications. Continued exploration of their unique characteristics will undoubtedly lead to new paradigms. In this context, we aim to provide readers with a comprehensive perspective on the use of luminescent BSDs and look forward to the next chapters in the exploration of this highly intriguing heterocyclic derivative.

## Conflict of Interests

The authors declare no conflict of interest.

## Data Availability

No primary research results and no new data were generated or analyzed as part of this review.
